# 

**Published:** 1880-10-20

**Authors:** 


### EDITORIALS AND MISCELLANEOUS.

*Henry Bill Publishing Company.*—See advertisement of the Henry Bill Publishing Company in this issue of the RECORD.

*Physicians' Visiting List for* 1881.—Lindsay & Blakeston's Visiting Dist for 1881 has been kindly presented by the publishers. Its reputation and usefulness have been fully established by long years of experience. It is certainly very useful to the practicing physician. Address, Lindsay & Blakestone, Philadelphia.

*Death of Dr. A. S. Fowler.*—We are much pained to have to record the death of Dr. A. S. Fowler, of Ringgold, Georgia, which occurred at his residence on the 7th of October, 1880, of consumption. Dr. A. T. Park, who communicated to us the sad intelligence, truly remarks, "That in the death of Dr. Fowler the citizens of Catoosa county have lost a good man and an eminent physician, and his brethren of the profession a genial companion, and a wise and safe counselor."

*Handsome Donation to Southern Medical College.*—That staunch and excellent establishment, William Wood & Co., 27 Great Jones Street, New York, generously presented to the Southern Medical College Library one dozen volumes, embracing their Library of Standard Medical Authors, as follows: Diseases of Children; Diseases of Liver, 3 vols.; Diseases of Intestines; Diseases of Nervous System, 2 vols.; Diseases of Women; Infant Feeding; Manual of Surgery; Rest and Pain; Materia Medica.

These works are approved and excellent, and constitute a compact little Library which the publishers are furnishing the profession at very low rates. Every practitioner would do well to obtain them.

### *VIRGINIA MEDICAL SOCIETY, HELD IN DANVILLE, VA., OCTOBER* 19, 1880.

The meeting was called to order by the President, Dr. Henry Latham. Dr. Edwards read the minutes of the last meeting, which were approved. Dr. Cunningham moved that the Association, after the President's Address, take a recess of ten minutes, and then go into the election of President. The Secretary, Dr. Edwards, moved that Dr. Payne, ex-Prisident of the North Carolina Medical Association, who was present, be extended the courtesies of the society. The President then said there was another distinguished medical gentleman present, Dr. Alban S. Payne, formerly of Virginia, but more recently the Professor of the "Theory and Practice of Medicine" in the Southern Medical College, Atlanta, whom we would be glad to see take his seat on this platform. The President then introduced these gentlemen, who responded in a few touching remarks to the compliment paid them.

WEDNESDAY NIGHT SESSION.—Dr. Hunter McGuire exhibited a case of resection of the entire upper half of the shaft of the tibia. Dr. McGuire had taken out, on other occasions during the war, one-half of the tibia in adults, the injuries being from gunshot wounds. Dr. Latham cited a similar case. Dr. Apperson then read a letter from Dr. Jones, descriptive of his own case, and asking that the society, after discussion, might advise him what to do. By request, Dr. Payne, of the Southern Medical College, Atlanta, gave his views on the treatment of the case reported by Dr. Apperson.

Dr. Dabney then read a valuable paper on the practical bearing of recent advance in cerebral Thermometry. Dr. White read an exceedingly interesting paper on the diseases of the eye.

After selecting Warrenton, Fauquer county, Virginia, as the next place of meeting, on motion of Dr. Edwards, the meeting adjourned.

N. S.

---

### *INTERNATIONAL MEDICAL CONGRESS, SEVENTH SESSION, LONDON,* 1881.

In a letter from Wm. McCormac, M.D., of London, it is stated that in past years the International Medical Congress has met in the following cities: The first meeting took place in Paris in 1867; the Congress met in Florence in 1869; then in Vienna in 1873; in Brussels in 1875; in Geneva in 1877; and last year, 1879, the Congress, as already stated, met in Amsterdam.

Her Majesty the Queen has most graciously given proof of her good-will towards the cause of Medical Science, and our efforts in its furtherance, by authorizing us to place the Congress under Her Royal patronage.

His Royal Highness the Prince of Wales has likewise shown the unvarying interest he takes in the progress of Medicine by according a similar favor.

The work of the Congress will be carried on in fifteen Sections. The days of the meeting will extend from Wednesday, the 3d, to Tuesday, the 9th of August, both days included. A Reception of Welcome will take place on the evening of August 2d.

The attendance of our countrymen from all parts of the United Kingdom, India and the Colonies will probably be large, and various circumstances make it probable that a large number of distinguished men from many countries will be attracted to England as our guests on the occasion of the Seventh Session of the Congress, and it is our desire to receive them with all cordiality and honor.

---

### *OPENING EXERCISES OF THE SOUTHERN MEDICAL COLLEGE.*

The opening exercises of the second session of the Southern Medical College took place on the 13th inst., in the Auditorium of the College. There was present a largely increased class, and a full audience of intelligent citizens, among whom were many ladies.

Rev, Dr. Hornady, one of the Trustees, opened the exercises with a brief and pertinent address, in which he announced the object of the founders to be nothing short of the establishment of a school of the very highest order, second to no school on the continent, and one whose diploma will be a source of honor and of pride to its fortunate possessor. On concluding his remarks, he introduced, as the orator of the occasion, Dr. R. C. Word, Professor of Physiology, who delivered the Introductory Address. The address on the subject of "Modern Advances in Knowledge," was received with marked attention and interest by the audience, and the Professor was warmly cougratulated by many.

P.

---

### *RECEIPTED.*

[Receipts not acknowledged privately are entered here.]

1880.—E. A. Anderson; W. T. McConnell; J. P. Stevens; A. J. Lewell; A. F. Durham; J. W. Bennett; H. S. Orme; E. L. Smith; C. M. Bold; T. B. Lanier (to Sept. 1880); J. B. Rutland; G. W. Wright.

1881.—A. H. Sellers.

